# Minor Surgery and Bandaging

**Published:** 1902-09

**Authors:** 


Minor Surgery and Bandaging. By Henry R. Wharton, M. D., Professor of Clinical Surgery in the Woman's Medical College; Surgeon to the Presbyterian Hospital, Philadelphia, etc. In one 12mo volume of 612 pages, with 509 illustrations, many of which are photographic. Fifth edition, enlarged and thoroughly revised. Philadelphia and New York: Lea Brothers & Co. 1902. [Price, $3.00 net.]

This work is designed primarily for the student and it seems to the reviewer to be well adapted to his needs. It contains a complete and well-illustrated chapter on bandaging, a full consideration of all procedures included under the head of minor surgery, chapters on fractures, dislocations, and a section on operations on the cadaver in which the principal operations in the domain of general surgery are both described and illustrated. The author is clear and concise in his descriptions, the illustrations, many of which are photographic, are to the point and, taken by and large, the book is thoroughly modern and altogether excellent. M. J. F.

